# Analysis of Giant‐Shell CdSe/CdS Quantum Dots via Analytical Ultracentrifugation Combined with Spectrally Resolved Photoluminescence

**DOI:** 10.1002/smtd.202401700

**Published:** 2025-01-12

**Authors:** Lisa M. S. Stiegler, K. David Wegner, Florian Weigert, Wolfgang Peukert, Ute Resch‐Genger, Johannes Walter

**Affiliations:** ^1^ Institute of Particle Technology (LFG) Department of Chemical and Biological Engineering (CBI) Friedrich‐Alexander‐Universität Erlangen‐Nürnberg (FAU) Cauerstraße 4 91058 Erlangen Germany; ^2^ Interdisciplinary Center for Functional Particle Systems (FPS) Friedrich‐Alexander‐Universität Erlangen‐Nürnberg (FAU) Haberstraße 9a 91059 Erlangen Germany; ^3^ Federal Institute for Materials Research and Testing (BAM) Division Biophotonics Richard‐Willstaetter‐Str. 11 12489 Berlin Germany

**Keywords:** analytical ultracentrifugation, multidimensional characterization, photoluminescence, quantum dots, sub‐nm size‐resolution

## Abstract

Knowledge of the structure–property relationships of functional nanomaterials, including, for example, their size‐ and composition‐dependent photoluminescence (PL) and particle‐to‐particle variations, is crucial for their design and reproducibility. Herein, the Angstrom‐resolution capability of an analytical ultracentrifuge combined with an in‐line multiwavelength emission detection system (MWE‐AUC) for measuring the sedimentation coefficient‐resolved spectrally corrected PL spectra of dispersed nanoparticles is demonstrated. The capabilities of this technique are shown for giant‐shell CdSe/CdS quantum dots (g‐QDs) with a PL quantum yield (PL QY) close to unity capped with oleic acid and oleylamine ligands. The MWE‐AUC PL measurements are calibrated and validated with certified fluorescence standards. The spectrally corrected and size‐dependent PL spectra of the g‐QDs derived from a single MWE‐AUC experiment are then analyzed and compared with the results of single‐particle spectroscopic studies, yielding the PL spectra, decay kinetics, and blinking behavior of individual g‐QDs. This study underlines the vast potential of MWE‐AUC with in‐line optical detection for the characterization of advanced nanomaterials with a complex structure.

## Graphical abstract

Analytical ultracentrifugation (AUC) is a powerful tool for studying the size–property relationships of nanomaterials in their native environment. A methodology based on AUC with in‐line multiwavelength emission detection is developed, enabling the measurement of the size‐resolved emission spectra of giant‐shell CdSe/CdS quantum dots. The results reveal core size dispersity, leading to the shifting and broadening of the emission band.

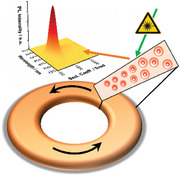

## 1 Introduction

Semiconductor nanocrystals, also termed quantum dots (QDs), are associated with size‐dependent absorption and emission features, very narrow symmetric emission bands, high photoluminescence (PL) quantum yields (QYs), and excellent photostability.^[^ ^1^ ^]^ These properties have paved the way for their application as luminescent reporters in bioimaging, biosensing, color multiplexing,^[^ ^2^ ^]^ optoelectronics,^[^ ^3^ ^]^ energy conversion, solid‐state lighting, and display technology.^[^ ^4^ ^]^ This has only been achievable after the year‐long development of suitable synthetic strategies,^[^ ^4^, ^5^ ^]^ for which the Nobel Prize in Chemistry was awarded in 2023.^[^ ^6^ ^]^ Today, most application‐relevant high‐quality II/VI, III/V, and IV/VI QDs present core/shell heterostructures capped with an organic ligand shell. The QD core is thereby passivated with a second semiconductor shell of controlled thickness with a larger bandgap to optimize the core's optical properties and stability against photodegradation and reduce its sensitivity to changes in the local QD environment. The ligand shell controls the QD colloidal stability and dispersibility and is relevant for further processing steps, such as the attachment of biomolecules and recognition moieties.^[^ ^7^ ^]^

Improving the synthesis and properties of QDs and elucidating the relationship between their structural properties, such as size, shape, and composition, and their absorption and PL features require suitable tools for their analysis, preferably in their native environment. Although ensemble measurements of QD batches can provide important information on their quality and photophysics, there is increasing interest in size‐resolved measurements of the optical properties of single QDs to distinguish and optimize the properties of individual QDs. Currently, the PL features of single QDs, such as PL spectra, PL decay kinetics, and their blinking behavior or fluorescence intermittency, are commonly evaluated via single‐particle spectroscopy. Such studies are typically performed with dried particles deposited on a solid substrate by spin coating or drop casting of very dilute QD dispersions. However, in the case of QDs, where coordinatively bound surface ligands are common, dilution can affect the integrity of the ligand shell and, thereby, the particles’ optical properties.^[^ ^8^ ^]^ This can result in reduced PL efficiency or QYs and may induce particle decomposition.^[^ ^9^ ^]^

The particle size distribution of QDs is usually obtained via a separate sizing technique, such as high‐resolution transmission electron microscopy (HR‐TEM), providing 2D information of single particles, or small‐angle X‐ray scattering (SAXS). The former requires counting a sufficiently high number of particles for statistically reliable results, while the latter measures ensembles of dispersed particles, thus relying on ill‐posed mathematical deconvolution to derive the size distribution. A notably promising alternative is analytical ultracentrifugation (AUC), which assesses samples directly in solution and yields results with excellent accuracy, resolution, and sample statistics. Considering this, AUC is widely used to study, for example, the properties of biological macromolecules,^[^ ^10^ ^]^ biopharmaceutical interactions,^[^ ^11^ ^]^ protein interactions,^[^ ^12^ ^]^ size–shape distributions,^[^ ^13^ ^]^ protein structure–function features,^[^ ^14^ ^]^ and therapeutic antibodies.^[^ ^15^ ^]^ The combination of the centrifugal separation of species according to their size, shape, and density with optical detection simplifies analytical procedures and opens new pathways. The potential of a custom‐built AUC device equipped with a multiwavelength absorption detection system (MWA‐AUC) for the study of nanomaterials has been recently demonstrated by us.^[^ ^16^ ^]^ Examples of areas of application include the determination of the size‐dependent absorption properties of polydisperse CdTe QDs,^[^ ^17^ ^]^ the size‐ and shape‐dependent extinction spectra of gold nanorods^[^ ^18^ ^]^ and bipyramids,^[^ ^19^ ^]^ and the size and composition distribution of gold–silver alloy nanoparticles.^[^ ^20^ ^]^

The combination of AUC with spectrally resolved PL detection is, however, largely underexplored, despite its vast potential in the analysis of luminescent nanomaterials and protein–protein interactions.^[^ ^21^ ^]^ We recently developed the first example of an AUC system with multiwavelength emission detection (MWE‐AUC),^[^ ^22^ ^]^ utilizing a 520 nm laser as the excitation light source and a charge‐coupled device (CCD) for detecting PL spectra in the range of 520–1000 nm. This setup was further improved by combining a mid‐focal length imaging spectrograph with a scientific‐grade electron‐multiplying charge‐coupled device (EMCCD) camera.^[^ ^23^ ^]^ Aiming to optimize the resolution of our MWE‐AUC system, we can now perform the high‐resolution spectral characterization of QDs. With this second‐generation MWE‐AUC setup, we explore herein its potential for measuring and quantifying the size‐ and composition‐dependent PL properties of fluorescent nanomaterials.

## 2 Results and Discussion

We selected ultrabright giant‐shell CdSe/CdS QDs (g‐QDs) with a relatively narrow size distribution as an application‐relevant sample, along with a PL QY close to unity and mono‐exponential PL decay kinetics. These g‐QDs exhibit a first excitonic absorption maximum at 614 nm and a PL maximum at 630 nm (full width at half maximum [FWHM] 31 nm/100 meV) in *n*‐hexane. Despite their nearly perfect PL features, single‐particle measurements have revealed differences in the PL spectra of single QDs.^[^ ^24^ ^]^ To explore the origin of these differences, possibly arising from QD‐to‐QD variations in the size of the QD core and/or shell thickness, we utilized the size and spectral resolution capabilities of our novel MWE‐AUC setup to analyze these g‐QDs directly in solution. This study is the first to provide insights into the nature of the PL heterogeneity of dispersed g‐QDs in their natural environment.

To compare the MWE‐AUC data with ensemble and single‐particle PL spectra measured with different instruments, we first calibrated our MWE‐AUC setup with certified fluorescence standards. This required considering the instrument‐specific wavelength‐dependent spectral responsivity of the detection channels of the different setups. This instrument‐specific quantity is determined by the wavelength‐dependent spectral responsivity of the detector and the wavelength‐dependent transmission of all optical components, such as fibers, mirrors, and filters, in the detection channel. The wavelength‐dependent spectral responsivity of the MWE‐AUC system was obtained with a set of certified fluorescence standards from BAM, with precisely known instrument‐independent emission spectra.^[^ ^25^ ^]^ In this regard, the certified BAM reference materials BAM‐F015 and BAM‐F017 were chosen, the PL spectra of which cover most of the visible wavelength region.

**Figure 1:**
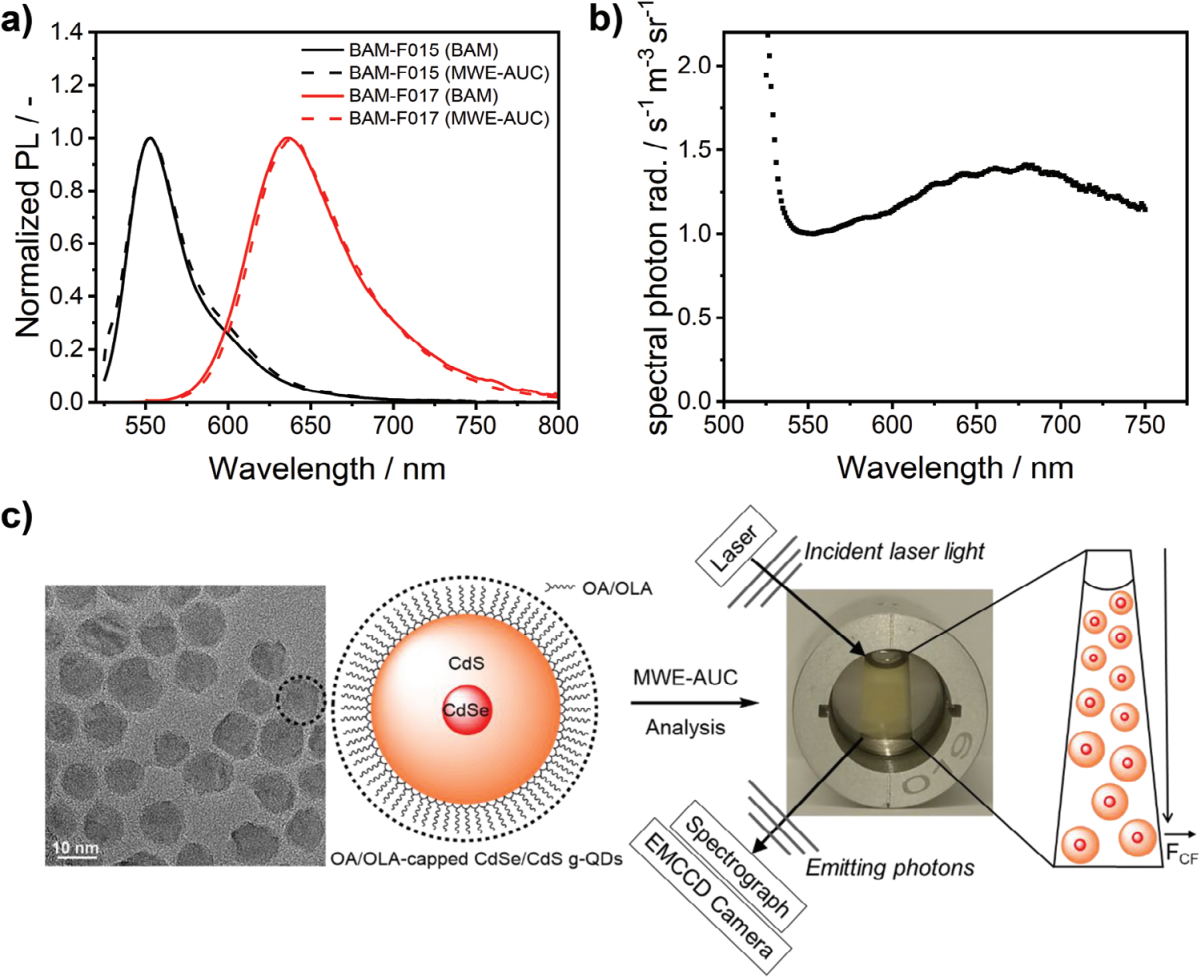
a) Normalized fluorescence spectra of BAM‐F015 (black) and BAM‐F017 (red) measured using a calibrated spectrometer (solid line) and the MWE‐AUC system without further corrections (dashed line). b) Spectral emission correction curve obtained from (a). c) Simplified schematic presentation of the oleic acid (OA)‐ and oleylamine (OLA)‐capped CdSe/CdS g‐QDs analyzed by MWE‐AUC, including a representative HR‐TEM image of the g‐QDs.

**Figure 2:**
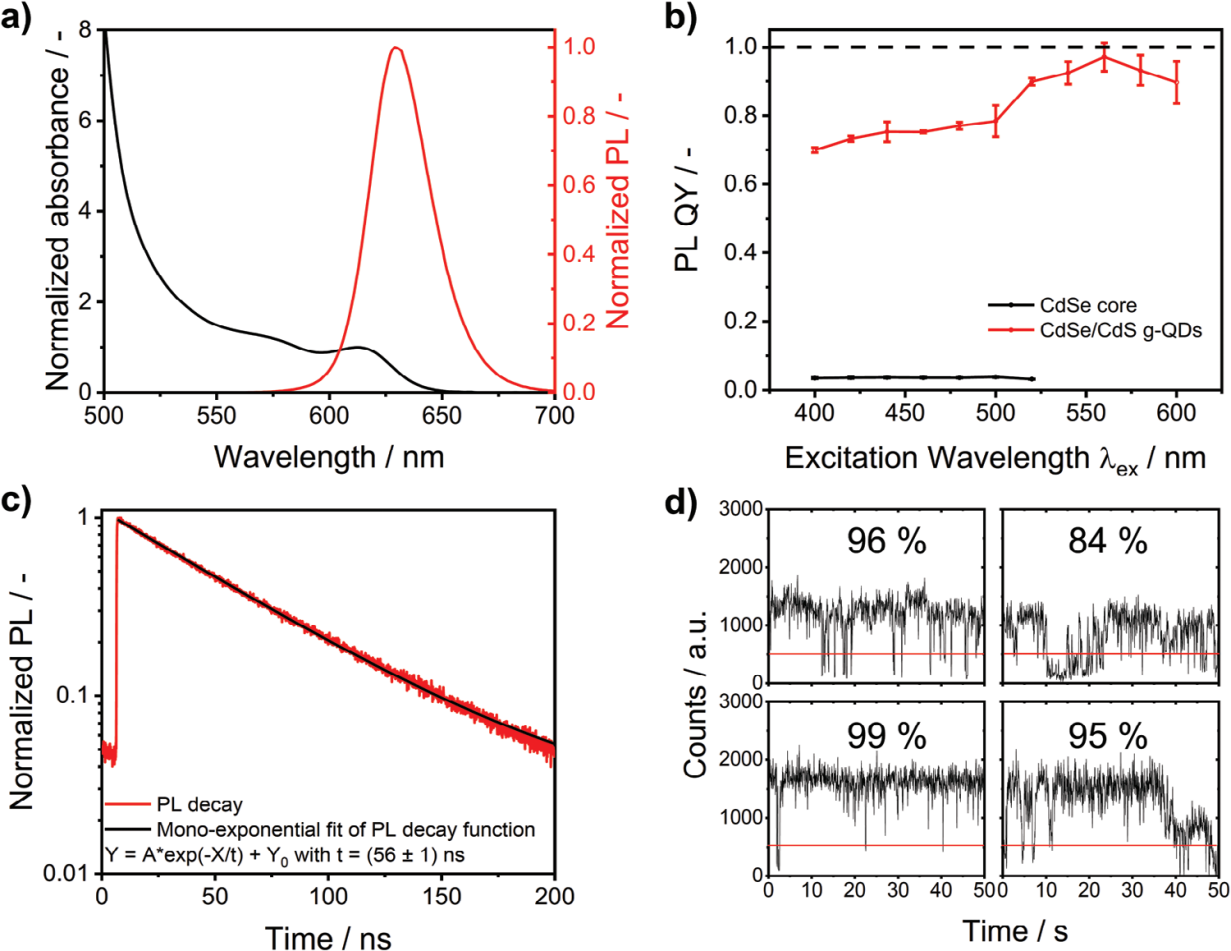
a) Absorbance and photoluminescence spectra of CdSe/CdS g‐QDs, b) photoluminescence quantum yields of the CdSe core and the CdSe/CdS g‐QDs excited at different wavelengths, c) normalized ensemble decay kinetics of CdSe/CdS g‐QDs fitted to a mono‐exponential function, and d) representative time traces of single CdSe/CdS g‐QDs with their calculated “ON” times.

The emission correction curve, equaling the instrument's wavelength‐dependent spectral responsivity, was obtained by dividing the uncorrected instrument‐dependent emission spectra (measured with the MWE‐AUC detection system) by the standards’ normalized spectrally corrected emission spectra (see **Figure** **1**a,b). The slight deviation in the PL spectrum of BAM‐F015 at 525 nm is ascribed to excitation with a 520 nm laser and possible contributions from scattered or reflected excitation photons. The emission correction curve shown in Figure 1b reveals discrepancies between the PL spectra measured with the MWE‐AUC system and the certified corrected emission spectra of the fluorescence standards beyond 600 nm. This highlights the need for spectral calibration and instrument‐independent PL spectra. The emission correction curve was then utilized to correct the fluorescence data derived from the sedimentation coefficient‐resolved PL measurements of the CdSe/CdS g‐QDs with our MWE‐AUC setup. The spectroscopic properties of the selected g‐QDs are provided in **Figure** **2** and Table S2 (Supporting Information).

**Figure 3:**
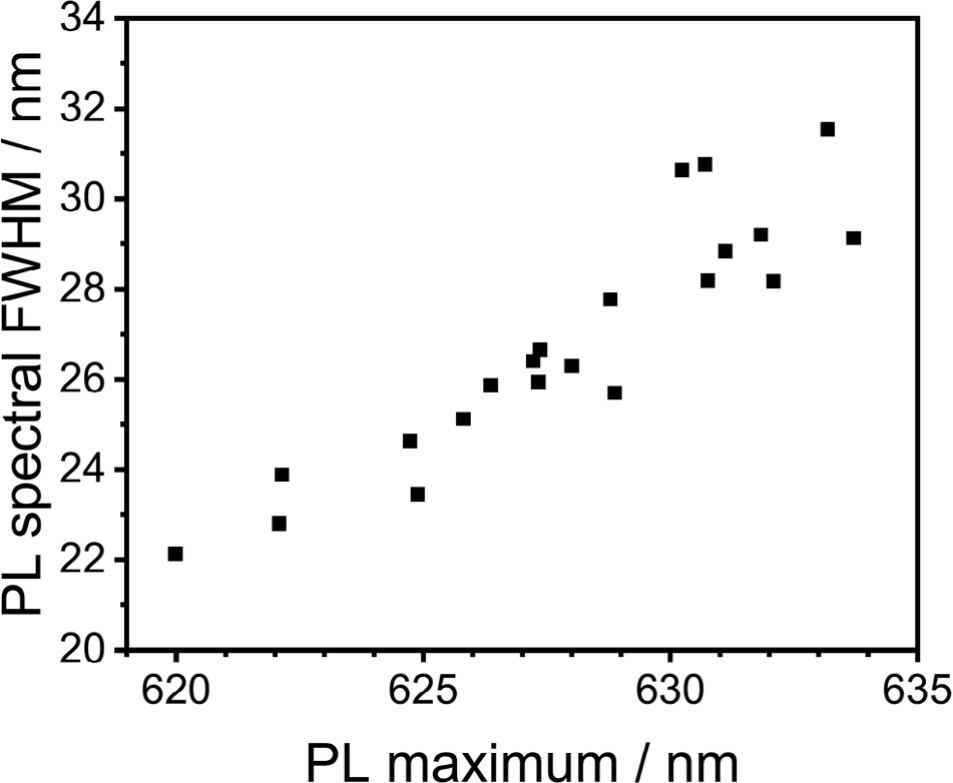
PL spectral width (FWHM) versus PL maximum of single g‐QDs excited at 405 nm.

The narrow first excitonic absorption peak and the narrow PL spectrum (see Figure 2a), together with previously reported TEM and SAXS data,^[^ ^24^ ^]^ indicate a very narrow size. One should note that larger QD size variations lead to a broader FWHM of the PL band. Furthermore, the PL QY is close to unity upon excitation at 520 nm, confirming the nearly complete absence of nonradiative recombination pathways due to a well‐passivated CdSe core (see Figure 2b). The PL decay kinetics, shown in Figure 2c, further reveal mono‐exponential behavior with an average lifetime of 56 ns. This is rare for QDs, which usually exhibit multi‐exponential PL decay kinetics due to defects and trap states within the bandgap. Single‐particle spectroscopy with dried QDs on a solid support confirmed the high quality of the CdSe/CdS g‐QDs; for example, time traces of single g‐QDs show “ON” times of almost 100 %, indicating their high chemical homogeneity, which is to be expected from the high PL QY (see Figure 2d). However, the PL spectra of single QDs still show some variations, that is, QD‐to‐QD differences in the spectral position of the PL maxima and FWHM (see **Figure** **3**). The origin of the observed spectral differences cannot be clarified solely by single‐particle spectroscopic studies because a direct correlation of the PL features of single QDs with their structural properties, such as size, is not possible.

**Figure 4:**
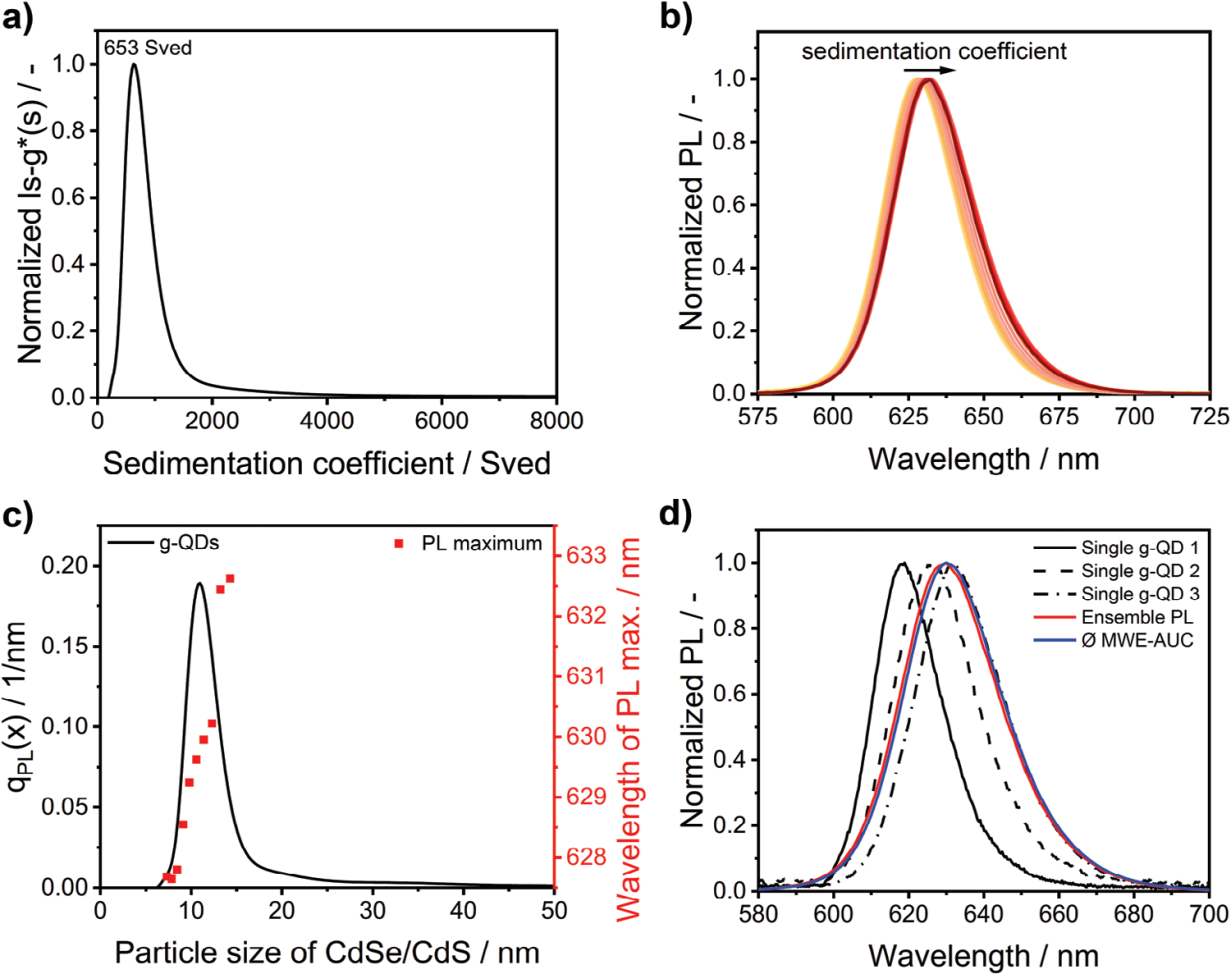
a) Sedimentation coefficient distribution derived from MWE‐AUC studies. b) QD PL spectra, with excitation at 520 nm, as a function of the sedimentation coefficient, ranging from 272 to 3320 Sved. c) Particle size distribution determined by MWE‐AUC, providing insights into the shift of the PL maxima of the CdSe/CdS g‐QDs. d) Comparison of the normalized PL spectra (solid lines) of single CdSe/CdS g‐QDs obtained via single‐particle spectroscopy, an averaged PL spectrum obtained by MWE‐AUC (dash‐dotted grey line), and an ensemble PL spectrum of the CdSe/CdS g‐QDs (dashed black line).

Here, assessing the sedimentation behavior of the entire CdSe/CdS g‐QD batch with our calibrated MWE‐AUC setup is advantageous. During the sedimentation process, the PL spectra of the g‐QDs between 570 and 730 nm were measured using a measuring cell with an optical path length of 3 mm (see Figure 1c). The obtained radial‐ and time‐dependent sedimentation profiles enabled the calculation of the sedimentation coefficient‐dependent PL spectra using direct boundary analysis. From the sedimentation coefficient distribution of the QDs at the respective PL maximum ≈630 nm (**Figure** **4**a), the sedimentation coefficient‐dependent PL spectra can be retrieved (Figure 4b); the corresponding 3D PL plot for Figure 4b is shown in Figure S1 (Supporting Information). These PL spectra were then corrected for the detection channel's wavelength‐dependent spectral responsivity via multiplication with the earlier obtained emission correction curve (see Figure 1c). The sedimentation coefficient distribution (Figure 4a) was then converted into the size distribution of the CdSe/CdS g‐QDs, shown in Figure 4c. Regarding the conversion based on the Stokes equation, we assumed a frictional ratio of 1 due to the particles’ nearly spherical shape derived from the TEM data analysis (Figure 1c).

**Figure 5:**
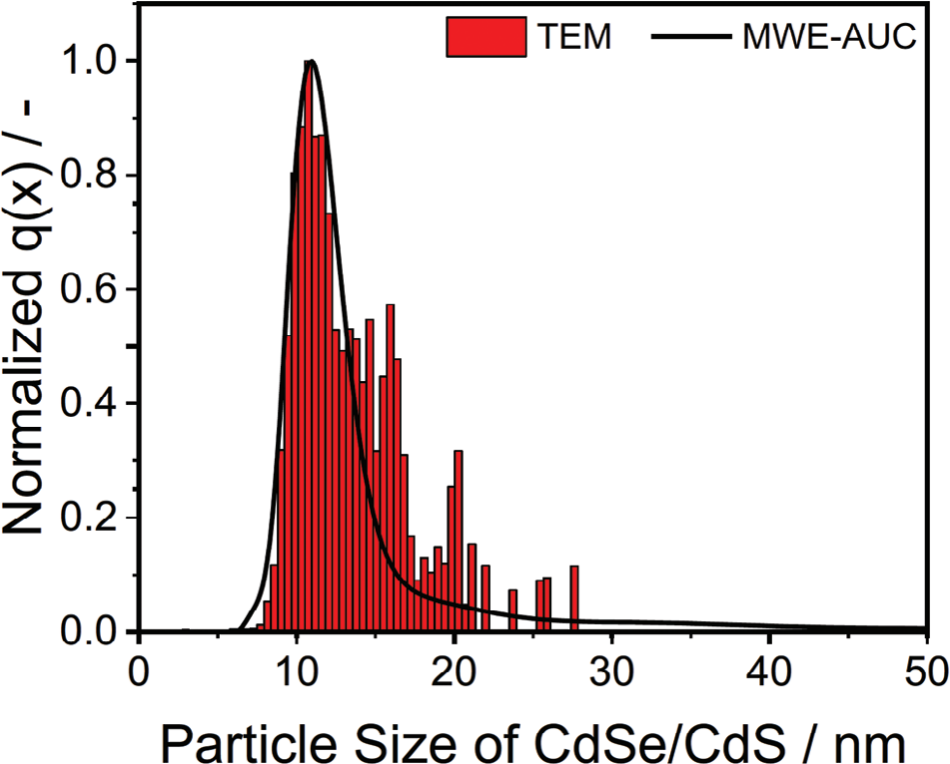
Size distribution of the CdSe/CdS g‐QDs obtained via TEM (histogram) and MWE‐AUC (black line).

The hydrodynamic diameter distribution including the organic ligand shell, consisting of oleic acid (OA) and oleylamine (OLA) molecules, is shown in Figure S2 (Supporting Information). In converting the sedimentation coefficient distribution to the size distribution, the following parameters were considered: the density of the CdSe/CdS g‐QDs (i.e., 4.85 g cm^−3^, as calculated from their volume ratio via HR‐TEM; see Supporting Information);^[^ ^24^ ^]^ the density of the solvated organic ligand shell, assuming a 1:1 ratio of the ligands OA and OLA; and the density of the solvent *n*‐hexane, in line with previous work,^[^ ^17^ ^]^ yielding an overall density of 0.74 g cm^−3^. Further parameters included in the calculation are the viscosity of the solvent (0.313 mPa·s) and the thickness of the organic ligand shell (estimated to be 1.9 nm).^[^ ^24^ ^]^ The reliability and accuracy of the resulting size distribution were confirmed by comparing with size measurements using TEM (see **Figure** **5**). The latter information, derived from 1400 counted QDs, was converted from a number‐ to a volume‐weighted distribution. The distributions show good correspondence, with modal size values of 10.7 and 11.0 nm for TEM and AUC, respectively. The MWE‐AUC data also match well with previously published SAXS measurements of the CdSe/CdS g‐QDs, yielding a number‐weighted mean QD diameter of 10.9 ± 0.6 nm.^[^ ^24^ ^]^

According to the analysis of the PL spectra of the CdSe/CdS g‐QDs, the QD PL maxima display a bathochromic shift of ≈5 nm with increasing QD size (see Figure 4c). This observation correlates well with the data obtained by single‐particle spectroscopy (see Figure 3). This finding confirms that the shift of the PL maximum arises from the size variation of the CdSe core and not from the defect or trap states within the bandgap of the g‐QDs. Furthermore, the tailing of the QD size distribution could point to the presence of a small fraction of agglomerated QDs. As follows from Figure S3a (Supporting Information), this leads to a shift of the PL maximum to shorter wavelengths, leveling off at lower values as the agglomerates are comprised of various primary particles with different sizes and emission properties. This leads to a loss of the size–emission maximum correlation. Simultaneously, the spectral width of the PL spectra (FWHM) increases from 29 to 32 nm until a decrease is observed in the region where QD agglomeration may become apparent (see Figure S3b, Supporting Information).

Figure 4d compares the normalized averaged PL spectrum, calculated from the sedimentation coefficient‐dependent PL spectra, with the normalized PL spectra of three representative single QDs. The ensemble PL spectrum peaks at 630 nm, and the averaged spectrum derived from the sedimentation coefficient‐dependent PL spectra peaks at 630 nm, while the slightly different single‐particle PL spectra exhibit maxima of 620, 627, and 634 nm. Notably, the PL band FWHM of 31 nm measured via MWE‐AUC matches the FWHM of the ensemble PL measurements. However, it is slightly broader than the FWHM determined for single dots, exhibiting values of 22.1, 26.7, and 29.1 nm. As the MWE‐AUC data can only resolve differences in nanoobject settling behavior, this observation suggests a size and inhomogeneity effect. Therefore, particles of the same sedimentation velocity consistently display a broader spectrum as they are broadened by particle‐to‐particle variations in the size, shape, surface, and composition. The slight differences between the MWE‐AUC‐derived PL data of the dispersed CdSe/CdS g‐QDs and the single‐dot PL spectra are ascribed to the different QD environments used in the measurements, where the single‐particle studies were conducted with dried QDs on a solid substrate. The effect of the environment on the PL properties of QDs has also been reported previously in the literature.^[^ ^26^ ^]^

## 3 Conclusion and Outlook

In summary, we demonstrated the Angstrom‐resolution power of a novel MWE‐AUC setup with an in‐line integrated PL detector, consisting of a spectrograph and an EMCCD camera, by measuring the sedimentation coefficient‐resolved PL spectra of high‐quality monodisperse giant core/shell CdSe/CdS QDs. Subsequently, the resulting PL spectra were compared with those of single QDs determined via single‐particle spectroscopy and PL ensemble measurements. The sedimentation coefficients, QD size populations, and respective PL spectra were then correlated.

The significant improvements of our MWE‐AUC setup have yielded a method to study the size‐ and composition‐dependent PL properties of nanomaterials. The method displays excellent accuracy; high resolution, allowing one to distinguish between size‐dependent spectra on the Angstrom scale; and excellent sample statistics, accounting for several billions of particles within a single measurement and a short measurement time of ≈60 min. This tool provides access to the particle size distributions of nanoparticles, such as QDs, and related variations in their PL characteristics without the tedious sample preparation and data evaluation procedures required by electron microscopy.

Future technical developments should focus on the combination of extinction and emission detection to determine the PL QY of photoluminescent QDs of different sizes via AUC. We believe that MWE‐AUC exhibits significant potential as a tool for quality and process control in nanotechnology, enabling researchers to assess the properties of luminescent nanoparticles and further develop and fine‐tune synthetic strategies.

## 4 Experimental Section

### Chemicals

The calibration standards BAM‐F015 and BAM‐F017 were provided by BAM. Ethanol was purchased from Honeywell.

### Giant Core/Shell CdSe/CdS QDs

The synthesis of the CdSe/CdS g‐QDs, consisting of a CdSe core, with a diameter of 4 nm, surrounded by a CdS shell comprising 11 CdS monolayers and capped/stabilized with OLA and OA ligand molecules, was performed by Dr. C. Wolters from Prof. H. Weller's group at the University of Hamburg.

### Transmission Electron Microscopy

As previously described in the literature,^[^ ^24^ ^]^ overview TEM images were obtained with a JEOL JEM‐2100 instrument (JEOL GmbH, Eching, Germany) operated at 200 kV. Image acquisition was carried out with a bottom‐mounted 4×4k CMOS camera (TemCam‐F416, TVIPS, Gauting, Germany). Data analysis was performed with ImageJ software, and the particle size and size distribution were determined by averaging 1400 particles. The HR‐TEM images were recorded with a FEI Titan 80–300 Berlin Holography Special TEM at 300 kV. The TEM samples were prepared by placing one drop of the highly dilute QD dispersion on a carbon‐coated copper grid (200 mesh, Electron Microscopy Sciences, Hatfield, PA), which was then air‐dried.

### Absorption and Emission Spectroscopy

Absorption spectra of the OA‐ and OLA‐stabilized CdSe/CdS g‐QDs dispersed in *n*‐hexane were recorded on a calibrated Cary 5000 UV–vis–NIR spectrometer (Varian, Agilent Technologies) with a spectral bandwidth and step size of 1 nm. The accuracy of the intensity and wavelength scale of this instrument is regularly controlled with certified absorption standards (Hellma GmbH). Steady‐state emission measurements were performed on a calibrated FSP 980 fluorescence spectrometer (Edinburgh Instruments) equipped with a xenon lamp (emission spectra) and a pulsed SC400‐PP supercontinuum fiber laser (Fianium) with a pulse width of 0.1 ns using time‐correlated single photon counting (TCSPC; fluorescence decay kinetics). So‐called magic‐angle conditions were applied (excitation and emission polarizers set to 0° and 54.7°, respectively) to render detected emission intensities independent of sample emission anisotropy.^[^ ^27^ ^]^ The emission spectra were corrected for solvent emission (blank correction) and the wavelength dependence of the instrument's spectral responsivity (spectral emission correction).^[^ ^28^ ^]^ The PL decay curves were fitted with a deconvolution fit using the FAST software (Edinburgh Instruments) and an instrument response function determined with a Ludox silica particle dispersion as a non‐emissive scatterer. All optical measurements were performed at room temperature (T  =  22 ± 2 °C) in 1 cm quartz cells (Hellma).

### Absolute Measurements of Photoluminescence Quantum Yields (PL QYs)

The absolute PL QY values of the CdSe/CdS g‐QDs were determined with a calibrated integrating sphere setup Quantaurus‐QY (Hamamatsu), previously evaluated by us,^[^ ^29^ ^]^ using long neck quartz cells (Hamamatsu). The absorbance at the excitation wavelength of 488 nm was kept below 0.1 to minimize inner filter effects and reabsorption, which would otherwise lead to an underestimation of the PL QYs.^[^ ^28^ ^]^

### Single‐Particle Measurements

Single‐particle PL measurements were performed with an inverted confocal laser scanning microscope using a UPLAPO100XO oil immersion objective, 100x, NA 1.4 (Olympus), mounted on an XYZ piezo stage. Sample excitation was performed with a 405 nm laser diode operated in the continuous (PL intensities and spectra) or pulsed mode (PL decay kinetics). Two sensitive single‐photon avalanche photodiodes (SPADs by MPD), arranged in a Hanbury Brown–Twiss geometry, were employed for the QD PL measurements utilizing a PicoQuant TimeHarp 260 Nano TCSPC Electronic system. Single‐particle PL spectra were collected using an Ocean Optics Peltier‐cooled CCD QE65 spectrometer with a fixed grating (350‐740 nm) coupled to an optical fiber. Detection of the excitation light and possible autofluorescence by the substrate was suppressed with a dichroic mirror and longpass filters. Regarding the optical studies of single CdSe/CdS g‐QDs, a highly dilute *n*‐hexane dispersion of the QDs was spin‐coated onto a glass cover slide. Subsequently, the single‐particle brightness, fluorescence intermittency via time traces (brightness over time, ON‐OFF blinking statistics), and PL decay kinetics were recorded from at least 30 single QDs. The single‐particle nature of the observed PL was confirmed by antibunching studies. The fluorescence intermittency of single QDs was obtained by recording PL time traces under continuous excitation, with the focus resting on the bright spots of the confocal image. To ensure measurements in the single‐exciton regime only and minimize the excitation intensity dependence of the power‐law blinking statistics, all experiments were performed with the lowest excitation power densities that still provided a reasonable signal‐to‐noise ratio.

### Multiwavelength Emission Analytical Ultracentrifugation

MWE‐AUC experiments were conducted with a modified preparative Optima L‐80K ultracentrifuge (Beckman Coulter) equipped with a custom‐made PL setup including a 520 nm excitation laser, as previously described.^[^ ^22^, ^23^ ^]^ The fluorescence standards in ethanol were prepared to achieve optical densities of 0.02 at 520 nm. The CdSe/CdS g‐QDs in *n*‐hexane were diluted to achieve an optical density of ≈0.25 at 520 nm. For the measurements, 200 µL of each solution or dispersion was transferred into a measuring cell equipped with a monosector titanium centerpiece with an optical path length of 3 mm (Nanolytics Instruments). Sedimentation velocity experiments were carried out with a rotor speed of 3000 rpm at a constant temperature of 20 °C. Additional experimental details are given in Table S1 (Supporting Information).

### Evaluation of MWE‐AUC Data

The sedimentation data of the QDs were evaluated at a detection wavelength of 625 nm with SEDFIT software (version 16.1c) using the ls‐g*(s) method,^[^ ^30^ ^]^ yielding the apparent sedimentation coefficient distribution with a resolution of 150 grid points. The data analysis included second‐derivative regularization, for which a confidence level (F‐ratio) of 0.95 was chosen. The meniscus position was fitted, and radially invariant and time‐invariant noise were corrected during the analysis. The x‐axis was selected as a grid with logarithmic spacing. The fluorescence spectra of the molecular fluorescence standards, which do not sediment, were derived from the raw data. The spectra were averaged over 10 scans between the radius positions of 6.5 and 7.0 cm. The sedimentation coefficient‐dependent PL spectra of the CdSe/CdS g‐QDs were obtained with a MATLAB program, utilizing the ls‐g*(s) method,^[^ ^31^ ^]^ that determines the apparent sedimentation coefficient distributions for all wavelengths. Similar to the SEDFIT software, the data analysis included second‐derivative regularization, for which a confidence level (F‐ratio) of 0.95 was chosen. In addition, the meniscus position was fitted, and radially invariant and time‐invariant noise were corrected during the analysis. Furthermore, the x‐axis was selected as a grid with logarithmic spacing. The resolution of the sedimentation coefficient distribution was set to 150 grid points, and that of the PL spectra was 1 nm; this yielded 150 sedimentation coefficients (in Sved), each with an associated PL spectrum.

## Conflict of Interest

The authors declare no conflict of interest.

## Supporting information



Supporting Information

## Data Availability

The data that support the findings of this study are openly available in [Zenodo] at [https://doi.org/10.5281/zenodo.14524874], reference number [14524874].

## References

[1] a) F. P. Garcia de Arquer , D. V. Talapin , V. I. Klimov , Y. Arakawa , M. Bayer , E. H. Sargent , Science 2021, 373, eaaz8541; 34353926 10.1126/science.aaz8541

[2] a) U. Resch‐Genger , M. Grabolle , S. Cavaliere‐Jaricot , R. Nitschke , T. Nann , Nat. Methods 2008, 5, 763; 18756197 10.1038/nmeth.1248

[3] a) Y. Shirasaki , G. J. Supran , M. G. Bawendi , V. Bulović , Nat. Photonics 2013, 7, 13;

[4] a) T. Lee , B. J. Kim , H. Lee , D. Hahm , W. K. Bae , J. Lim , J. Kwak , Adv. Mater. 2022, 34, 2106276; 10.1002/adma.202106276 34706113

[5] J. J. Li , Y. A. Wang , W. Z. Guo , J. C. Keay , T. D. Mishima , M. B. Johnson , X. G. Peng , J. Am. Chem. Soc. 2003, 125, 12567. 14531702 10.1021/ja0363563

[6] K. D. Wegner , U. Resch‐Genger , Anal. Bioanal. Chem. 2024, 416, 3283. 38478110 10.1007/s00216-024-05225-9 PMC11106203

[7] P. Reiss , M. Protière , L. Li , Small 2009, 5, 154. 19153991 10.1002/smll.200800841

[8] M. Grabolle , M. Spieles , V. Lesnyak , N. Gaponik , A. Eychmuller , U. Resch‐Genger , Anal. Chem. 2009, 81, 6285.

[9] O. Dukhno , F. Przybilla , V. Muhr , M. Buchner , T. Hirsch , Y. Mely , Nanoscale 2018, 10, 15904. 30106079 10.1039/c8nr03892a

[10] G. B. Edwards , U. M. Muthurajan , S. Bowerman , K. Luger , Curr. Protoc. Mol. Biol. 2020, 133, e131. 33351266 10.1002/cpmb.131 PMC7781197

[11] J. P. Gabrielson , K. K. Arthur , M. R. Stoner , B. C. Winn , B. S. Kendrick , V. Razinkov , J. Svitel , Y. Jiang , P. J. Voelker , C. A. Fernandes , R. Ridgeway , Anal. Biochem. 2010, 396, 231. 19782040 10.1016/j.ab.2009.09.036

[12] a) P. Schuck , Anal. Biochem. 2003, 320, 104; 12895474 10.1016/s0003-2697(03)00289-6

[13] P. H. Brown , P. Schuck , Biophys. J. 2006, 90, 4651. 16565040 10.1529/biophysj.106.081372 PMC1471869

[14] C. Y. Chou , Y. H. Hsieh , G. G. Chang , Methods 2011, 54, 76. 21087667 10.1016/j.ymeth.2010.11.002 PMC7128498

[15] S. Uchiyama , M. Noda , E. Krayukhina , Biophys. Rev. 2018, 10, 259. 29243091 10.1007/s12551-017-0374-3 PMC5899733

[16] a) J. Walter , K. Löhr , E. Karabudak , W. Reis , J. Mikhael , W. Peukert , W. Wohlleben , H. Cölfen , ACS Nano 2014, 8, 8871; 25130765 10.1021/nn503205k

[17] E. Karabudak , E. Brookes , V. Lesnyak , N. Gaponik , A. Eychmuller , J. Walter , D. Segets , W. Peukert , W. Wohlleben , B. Demeler , H. Cölfen , Angew. Chem., Int. Ed. 2016, 55, 11770. 10.1002/anie.201603844 PMC5148131 27461742

[18] S. E. Wawra , L. Pflug , T. Thajudeen , C. Kryschi , M. Stingl , W. Peukert , Nat. Commun. 2018, 9, 4898. 30464237 10.1038/s41467-018-07366-9 PMC6249260

[19] U. Frank , D. Drobek , A. Sanchez‐Iglesias , S. E. Wawra , N. Nees , J. Walter , L. Pflug , B. Apeleo Zubiri , E. Spiecker , L. M. Liz‐Marzan , W. Peukert , ACS Nano 2023, 17, 5785. 36920091 10.1021/acsnano.2c12257

[20] a) P. Cardenas Lopez , M. J. Uttinger , N. E. Traoré , H. A. Khan , D. Drobek , B. Apeleo Zubiri , E. Spiecker , L. Pflug , W. Peukert , J. Walter , Nanoscale 2022, 14, 12928; 36043498 10.1039/d2nr02633c

[21] a) H. Zhao , M. L. Mayer , P. Schuck , Anal. Chem. 2014, 86, 3181; 24552356 10.1021/ac500093m PMC3988680

[22] S. E. Wawra , G. Onishchukov , M. Maranska , S. Eigler , J. Walter , W. Peukert , Nanoscale Adv. 2019, 1, 4422. 36134402 10.1039/c9na00487d PMC9419176

[23] V. Lautenbach , G. Onishchukov , S. E. Wawra , U. Frank , L. Hartmann , W. Peukert , J. Walter , Nanoscale Adv. 2024, 6, 2611. 38752146 10.1039/d3na00980g PMC11093262

[24] F. Weigert , A. Muller , I. Hausler , D. Geissler , D. Skroblin , M. Krumrey , W. Unger , J. Radnik , U. Resch‐Genger , Sci. Rep. 2020, 10, 20712. 33244030 10.1038/s41598-020-77530-z PMC7692488

[25] a) U. Resch‐Genger , W. Bremser , D. Pfeifer , M. Spieles , A. Hoffmann , P. C. DeRose , J. C. Zwinkels , F. Gauthier , B. Ebert , R. D. Taubert , C. Monte , J. Voigt , J. Hollandt , R. Macdonald , Anal. Chem. 2012, 84, 3889; 22376085 10.1021/ac2034503

[26] D. E. Gómez , J. v. Embden , P. Mulvaney , Appl. Phys. Lett. 2006, 88, 154106.

[27] D. Geissler , C. Wurth , C. Wolter , H. Weller , U. Resch‐Genger , Phys. Chem. Chem. Phys. 2017, 19, 12509. 28470291 10.1039/c7cp02142a

[28] C. Würth , M. Grabolle , J. Pauli , M. Spieles , U. Resch‐Genger , Nat. Protoc. 2013, 8, 1535. 23868072 10.1038/nprot.2013.087

[29] C. Würth , C. Lochmann , M. Spieles , J. Pauli , K. Hoffmann , T. Schüttrigkeit , T. Franzl , U. Resch‐Genger , Appl. Spectrosc. 2010, 64, 733. 20615286 10.1366/000370210791666390

[30] P. Schuck , P. Rossmanith , Biopolymers 2000, 54, 328. 10935973 10.1002/1097-0282(20001015)54:5<328::AID-BIP40>3.0.CO;2-P

[31] J. Walter , T. Thajudeen , S. Süß , D. Segets , W. Peukert , Nanoscale 2015, 7, 6574. 25789666 10.1039/c5nr00995b

