# Supplementary material for: Analysis of Giant‐Shell CdSe/CdS Quantum Dots via Analytical Ultracentrifugation Combined with Spectrally Resolved Photoluminescence
Source: Small Methods. 2025 Jan 12;10(2):2401700. doi: 10.1002/smtd.202401700 (PMC12825350; doi:10.1002/smtd.202401700)
Supplement: Supplementary file 1 — Supporting Information [file SMTD-10-2401700-s001.docx]

Supporting Information

Analysis of Giant-Shell CdSe/CdS Quantum Dots via Analytical Ultracentrifugation Combined with Spectrally Resolved Photoluminescence

Lisa M. S. Stiegler,^[a],[b]#^ K. David Wegner,^[c]#^ Florian Weigert,^[c]^ Wolfgang Peukert,^[a],[b]^ Ute Resch-Genger,^*[c]^ and Johannes Walter^*[a],[b]^

**Additional experimental details on the MWE-AUC settings**

**Table S1.** MWE-AUC settings. Software MWE 3.08c – OS BETA.

| **Parameters to set** | **Selected option** |
| --- | --- |
| Temperature | 20 °C |
| Rotor | 8-hole |
| Rotor speed | 3000 rpm |
| Scan range | 5.8–7.2 cm |
| Step | 50 µm |
| Central wavelength | 730 nm |
| Grating | 150 g/mm |
| Record from | 460–1000 nm |
| Average scans | 1 |
| Time between scans | 10 s |
| Number of scans | 109 |
| Exposure time | 150 ms |

**Details on the ensemble and single particle photoluminescence measurements**

**Table S2.** First excitonic absorption band (Abs. max.), photoluminescence maximum (PL max.), PL emission band fullwidth at half maximum (FWHM), PL lifetime (PL LT), and PL quantum yield (PL QY) of CdSe/CdS g-QDs obtained via ensemble versus single-particle measurements.

| Measurement mode | Abs. max (nm) | PL max. (nm) | PL band FWHM (nm, meV) | PL LT (ns) | PL QY (%) |
| --- | --- | --- | --- | --- | --- |
| Ensemble | 614 | 630 | 31, 100 | 56 | 93 |
| Single-particle (average) |  | 628±4 | 12±1,36 | 73 |  |

**Calculation of the mixing density of the g-QDs**


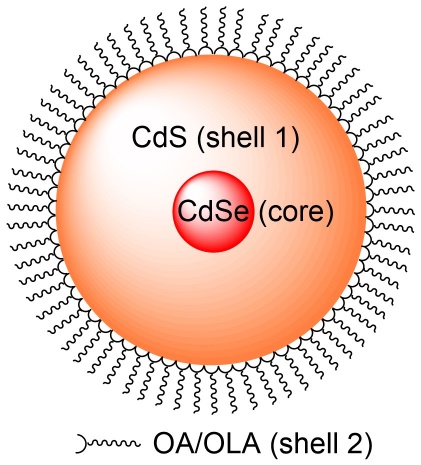
CdSe/CdS 11 ML (d. 3.48 nm/d. 11.16 nm)

$V_{QD (core+shell 1)}=\frac{4}{3}*\pi*\left( 5.58 nm \right)^{3}=727.765 {nm}^{3}$

$V_{core}=\frac{4}{3}*\pi*{(1.74 nm)}^{3}$ $=22.067 {nm}^{3}$

$V_{shell 1}=$ $V_{QD}- V_{core} =705.696 {nm}^{3}$

$\frac{V_{core}}{V_{shell 1}}=0.03 =3:100$

$\rho\left( CdSe/CdS 11 ML \right)=4.849 g/{cm}^{3}$

**Scheme S1.** Structure of the g-QD including the core (CdSe), shell 1 (CdS), and shell 2 (OA/OLA). Adjacent: Calculation of the mixed density of the core and shell 1 of the g-QDs.

**Sedimentation coefficient-dependent PL spectra**


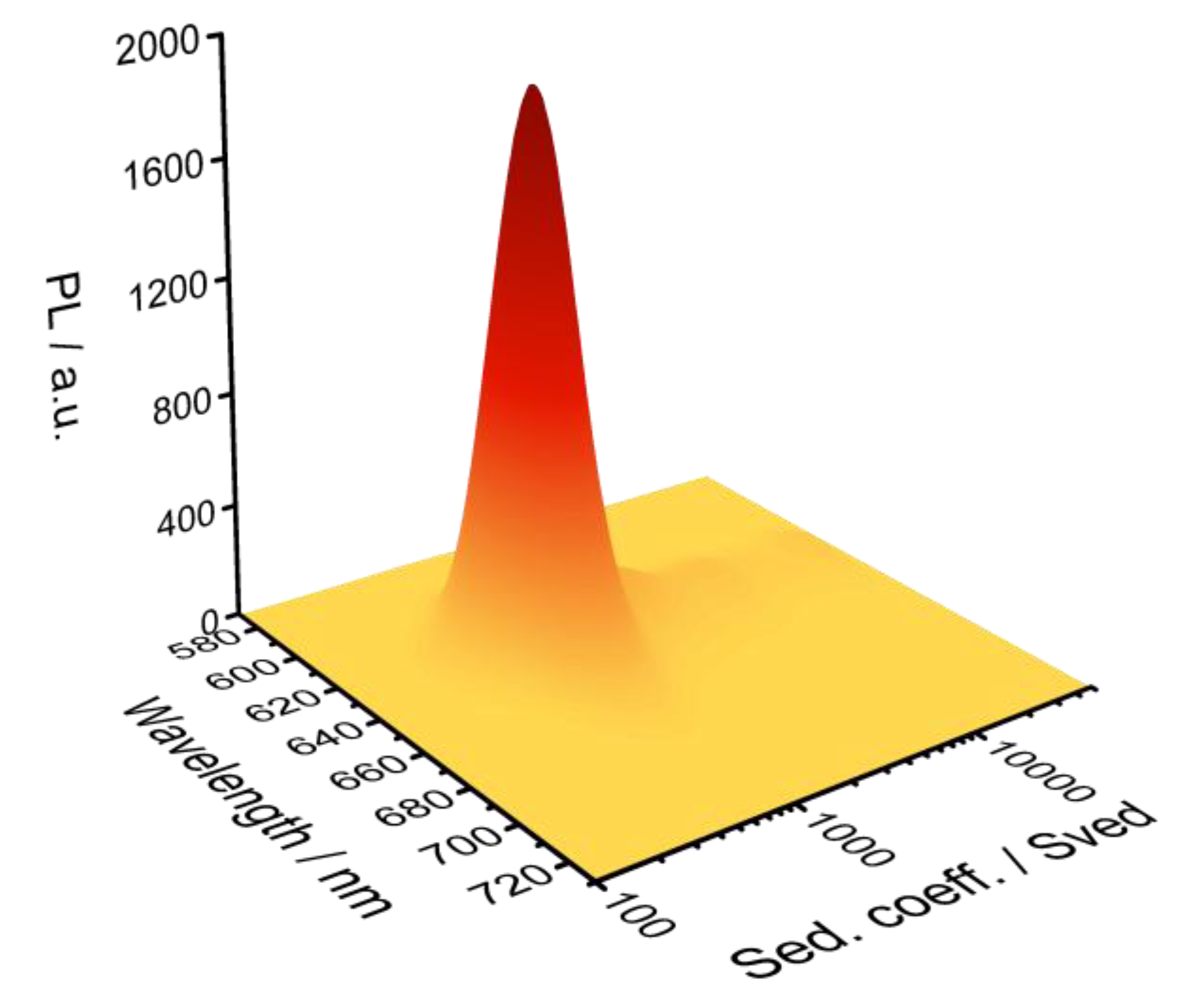


**Figure S1.** PL intensity as a function of wavelength and sedimentation coefficient.

**Size distribution of the CdSe/CdS g-QDs obtained by MWE-AUC**


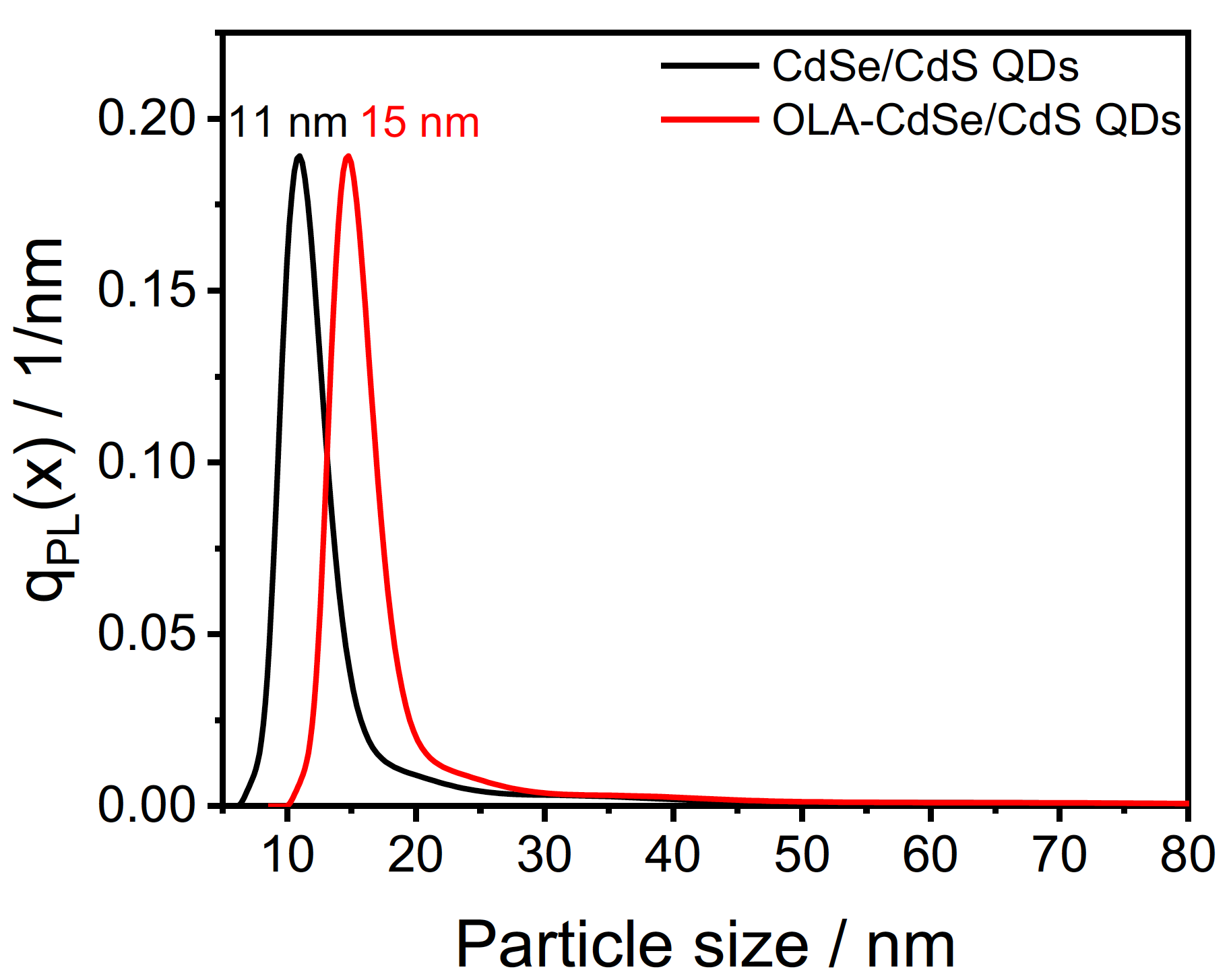


**Figure S2.** Size distribution of CdSe/CdS QDs without (black line) and with (red line) OA/OLA ligand shell.

**Comparison of size distributions of g-CdSe/CdS QDs obtained by MWE-AUC**


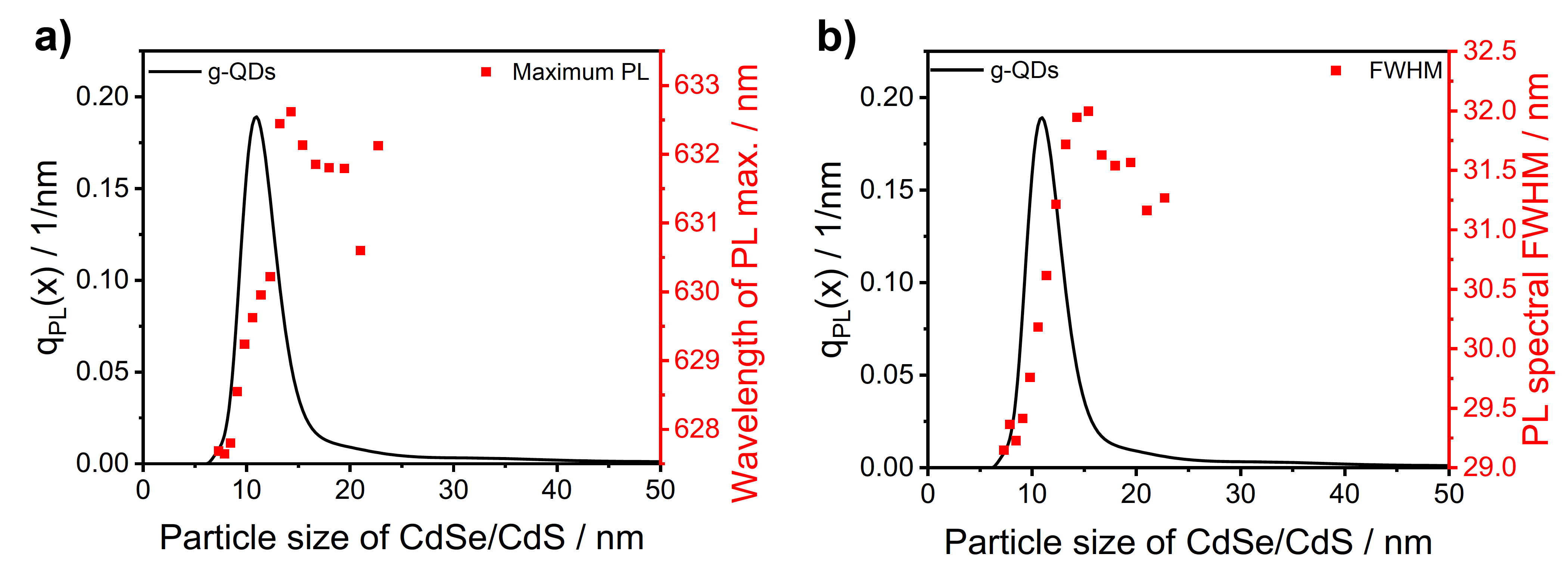


**Figure S3.** Particle size distributions, providing insights into **(a)** the particle size-induced shifts of the PL maximum and **(b)** the FWHM of the PL spectra of the CdSe/CdS g-QDs in *n*-hexane.
